# A new *P. falciparum* gametocyte drug screening assay based on pLDH detection

**DOI:** 10.1186/1475-2875-11-S1-O34

**Published:** 2012-10-15

**Authors:** Sarah D’Alessandro, Nicoletta Basilico, Yolanda Corbett, Silvia Parapini, Francesco Silvestrini, Koen Dechering, Tiziana Bianchi, Paola Verducci, Robert Sauerwein, Pietro Alano, Donatella Taramelli

**Affiliations:** 1 Dipartimento di Scienze Farmacologiche e Biomolecolari, Università di Milano, Milan, 20134, Italy; 2 Dipartimento di Scienze Biomediche, Chirurgiche eOdontoiatriche, Università di Milano, Milan, 20133, Italy; 3 Dipartimento di Malattie lnfettive, Parassitarie, Immunomediate, Istituto Superiore di Sanità, Roma, 00161, Italy; 4 TroplQ Health Sciences, Nijmegen, 6525 GA, The Netherlands; 5 AVIS Comunale di Milano, Milan, 20133, Italy; 6 Department of Medical Microbiology, Radboud University Nijmegen Medical Center, Nijmegen, 6525 GA, The Netherlands

## 

*Plasmodium* gametocytes (GCT) have recently been proposed as a crucial target for the development of new antimalarials in order to achieve malaria elimination and eventually eradication. At present, however, a widely accepted and routinely used screening method for potential gametocytocidal drugs does not exist. The aim of our work was to adapt the parasite lactate dehydrogenase (pLDH), already standardised for drug screening on asexual stages, to measure gametocyte drug sensitivity. In clinics the GCT- pLDH, which is present during all the five stages of gametocyte development, can be measured with good sensitivity through OptiMAL, an immunochromatographic diagnostic test. The pLDH assay is fast, simple, not expensive and does not require complex equipment or special waste disposal. It can be applied to field isolates since transgenesis is not needed.

Gametocytogenesis of two different strains of *P. falciparum*, 3D7 and NF54, was induced *in vitro* using a standardized protocol, asexual parasites were removed by N-acetylglucosamine treatment, and GCT were seeded in 96well plates. A linear correlation between the percentage of gametocytemia, microscopically counted by Giemsa staining, and the optical density, measured spectroscopically by pLDH assay, was demonstrated. A good signal to noise ratio was obtained with the pLDH assay, and the Z’factor was calculated as indicator of the robustness of the method. Our data also indicate that GCT have a pLDH activity higher than asexual parasites.

GCT were treated for 48-72h with primaquine, the gold standard against mature gametocytes *in vivo*, which was used to validate most of the GCT screening methods in literature; dihydroartemisinin, active on young GCT; and methylene blue, an old antimalarial recently characterised also for its anti-GCT activity. Finally, epoxomicin was tested since its strong gametocytocidal effect has been recently reported. Dose-response curves were obtained with all the four drugs. However, some discrepancies were observed between the Giemsa staining and the pLDH detection at high concentrations of the drugs, suggesting that morphological abnormalities, detected microscopically, precede the decay of pLDH activity in drug-treated GCT. In order to better understand these observations, we prolonged the treatment for further 72h. This extra-incubation period allowed us to calculate, from the pLDH assay, the IC_50_ (as the 50% inhibition compared to control untreated GCT) of the tested compounds, which were comparable to those obtained by Giemsa staining.

These results demonstrate the feasibility of pLDH assay to measure GCT content in culture. Although more specific probes for GCT viability need to be standardized for measuring stage-specific drug activity, pLDH can be used as the first, fast and cheap screening method to find potential gametocitocydal drugs.

